# Efficacy of low-dose fipronil bait against blacklegged tick (*Ixodes scapularis*) larvae feeding on white-footed mice (*Peromyscus leucopus*) under simulated field conditions

**DOI:** 10.1186/s13071-021-04930-z

**Published:** 2021-09-07

**Authors:** David M. Poché, Kelsey Dawson, Batchimeg Tseveenjav, Richard M. Poché

**Affiliations:** Genesis Laboratories, Inc., Wellington, CO USA

**Keywords:** *Borrelia burgdorferi *sensu stricto, Blacklegged ticks, *Ixodes scapularis*, White-footed mice, *Peromyscus leucopus*, Fipronil bait, Acaricides, Systemic insecticides, Vector control, Simulated field conditions

## Abstract

**Background:**

Lyme disease, caused primarily by *Borrelia burgdorferi* sensu stricto, is the most prevalent vector-borne disease in the United States. Treatment of rodent pathogen reservoirs with an oral acaricide may suppress the production of infected host-seeking ticks posing a risk for human infection. A previous study showed that an oral fipronil bait effectively controlled larval *Ixodes scapularis* ticks on white-footed mice (*Peromyscus leucopus*) up to 15 days post-bait exposure. The present study expands upon this finding by exposing group-housed white-footed mice to fipronil bait under simulated field conditions prior to tick infestation.

**Methods:**

Mice (*n* = 80) were housed in groups of 10 within large enclosures and offered a choice between fipronil bait within a commercial bait station and an alternative diet. The mice were assigned to two treatment groups and two control groups to undergo bait exposure durations of either 24 h (reduced) or 168 h (extended). Groups were further differentiated by the time point post-bait exposure when larval ticks were applied to mice within feeding capsules (reduced day 1, day 15; extended day 21, day 35). For 4 days post-tick introduction, attached larvae were observed by microscopy and replete larvae were recovered. Replete larvae were monitored for molting success. Plasma was collected from all treatment group mice to obtain fipronil plasma concentrations (CP).

**Results:**

The fipronil bait (0.005% fipronil) was palatable and controlled larval ticks on white-footed mice when presented under simulated field conditions. Efficacy in preventing attached larvae from feeding to repletion was 100% (day 1), 89.0% (day 15), 85.8% (day 21), and 75.2% (day 35). When also considering molting success, the fipronil bait prevented 100% (day 1), 91.1% (day 15), 91.7% (day 21), and 82.5% (day 35) of larvae attaching to mice from molting. The mean CP per mouse was 191.5 ng/ml (day 1), 29.4 ng/ml (day 15), 10.6 ng/ml (day 21), and 1.0 ng/ml (day 35).

**Conclusions:**

The results suggest that fipronil bait will be consumed by white-footed mice in the presence of an alternative diet, and effectively control larval ticks on treated mice. A field trial is needed to confirm the results of this study. Low-dose fipronil bait may provide a cost-effective means of controlling blacklegged ticks to be integrated into tick management programs.

**Graphical Abstract:**

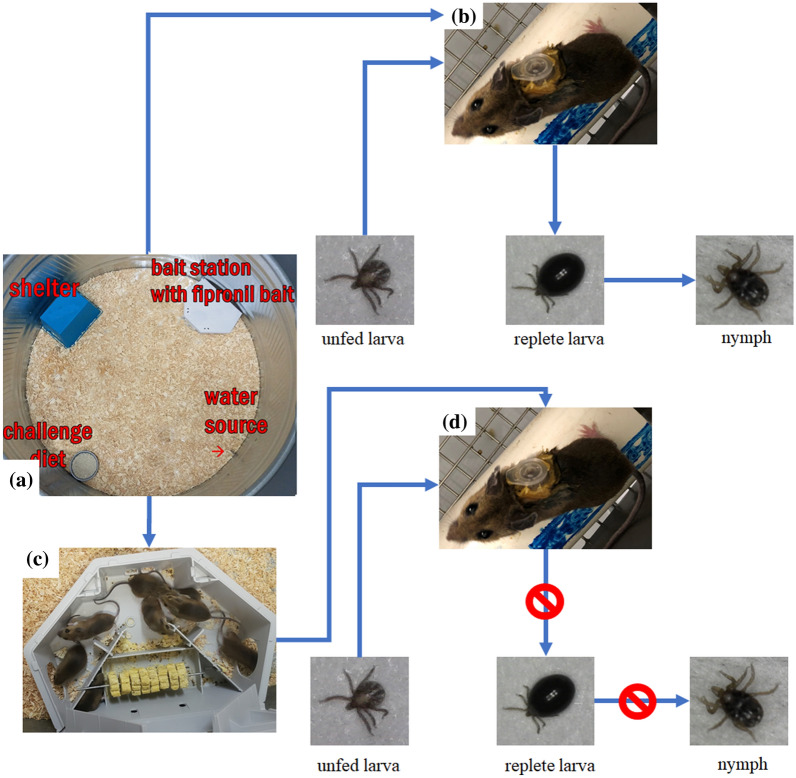

**Supplementary Information:**

The online version contains supplementary material available at 10.1186/s13071-021-04930-z.

## Background

Lyme disease, caused primarily by *Borrelia burgdorferi* sensu stricto (s.s.), is the most prevalent vector-borne disease in the United States [1]. The geographical distributions of both tick vectors and human Lyme disease cases have expanded, and the numbers of reported human cases have steadily increased since standardized surveillance began in 1991 [2, 3]. The Centers for Disease Control and Prevention (CDC) estimates around 500,000 Lyme disease infections each year with the majority of cases occurring in the Midwest and Northeastern USA [4, 5].

The blacklegged tick (*Ixodes scapularis*) is the primary vector of *B. burgdorferi* s.s. in the Midwest and Northeast [6] and its range in the eastern USA is expanding [3]. Climate change may contribute to this geographical expansion [7], as blacklegged ticks prefer warm temperatures and dry to mesic deciduous forests on alfisol-type sandy or loam soils [8]. The blacklegged tick has been documented in at least 45.7% of the continental USA and blacklegged ticks and the western blacklegged tick (*Ixodes pacificus*) are now present in at least 49.2% of the counties in the continental United States, marking a 44.7% expansion in range since 1998 [3].

Blacklegged tick larvae hatch uninfected and can acquire *B. burgdorferi* s.s. after feeding on an infected reservoir host [9]. The white-footed mouse (*Peromyscus leucopus*) is a primary reservoir for *B. burgdorferi* s.s. in the Midwest and Northeastern USA regions where Lyme disease is most prevalent [10, 11]. While immature blacklegged ticks do feed on a variety of hosts, many of these species are relatively poor reservoirs for the Lyme disease spirochete [11]. Nymphs are primarily responsible for the transmission of *B. burgdorferi* s.s. to humans, and the prevalence of infection in *I. scapularis* nymphs is generally in the range of 15 to 25% in the Midwest and Northeast [12]. The risk of human exposure to Lyme disease spirochetes is correlated with the abundance of infected host-seeking nymphs. Although several different intervention strategies targeting blacklegged ticks have been utilized over the past few decades, there has been an overall lack of success in reducing instances of human Lyme disease [13, 14]. Oral acaricides offered to rodent reservoirs of *B. burgdorferi* s.s. present a promising tick suppression strategy that could be integrated into tick management programs. Control of ticks infesting rodents also may disrupt the enzootic transmission cycle, further reducing the density of infected host-seeking ticks [14].

Fipronil is a phenylpyrazole that interferes with the central nervous system in arthropods through the blockage of GABA-gated and glutamate-gated chloride channels [15]. When used in an oral acaricide bait, fipronil acts systemically, being consumed by ticks during blood-feeding [16]. A recent laboratory study indicated that a low-dose fipronil bait (0.005%) presented orally to white-footed mice for 48 h could control 100% of blacklegged tick larvae blood-feeding at days 1, 9 and 15 post-exposure [17]. These results provided early evidence suggesting a fipronil-based oral acaricide bait could be a useful addition to integrated tick management programs.

While the above study was useful in establishing proof of concept, additional studies could provide further insights into the use of fipronil bait under field conditions. The fipronil bait utilized in the above experiment [17] was presented in open containers for 48 h in a no-choice test to individually housed mice. Under field conditions, federal regulations may require bait to be presented in tamper-resistant bait stations. Additionally, contrary to the laboratory experiment, wild mice would have access to alternative food sources, which could impact consumption, and individual wild mice would feed on the fipronil bait at different frequencies and durations. The results of the previous laboratory work indicate 100% efficacy to be obtained up to 15 days post-exposure, suggesting that adequate control could potentially be obtained several weeks after application. This assumption is supported by field research in which fleas parasitizing wild rodents have been controlled for several weeks and months post-application of a granular bait containing the same concentration of fipronil as the above study [18–21]. Addressing the above limitations could aid in developing more robust tick management strategies.

## Methods

**Fig. 1:**
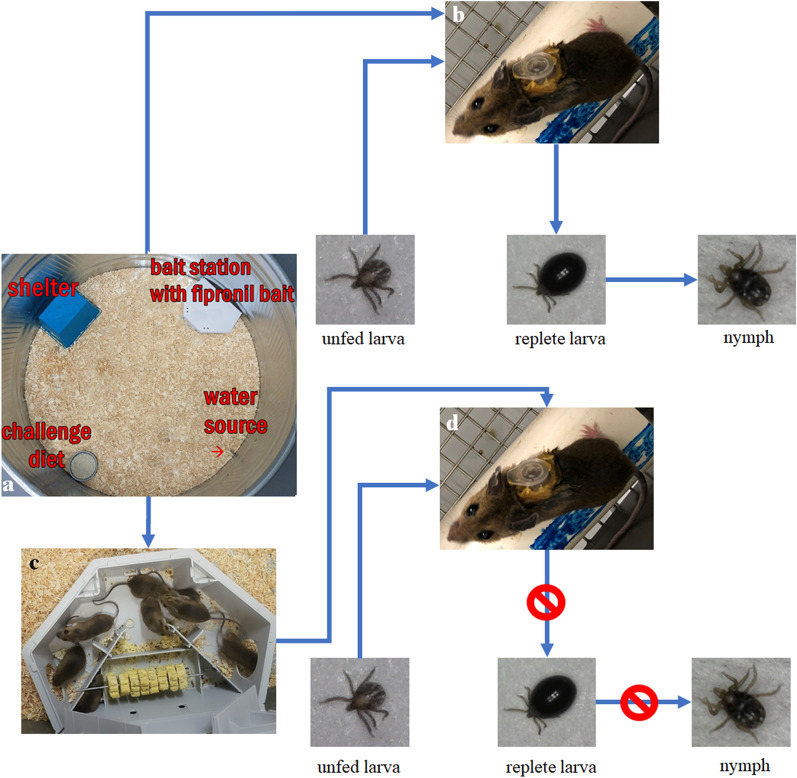
Conceptual diagram for the experimental design of the study. **a** Mice group-housed in a large enclosure and presented with bait station, alternative diet, water source, and shelter. **b** Control group mice received no fipronil bait prior to being transferred to the insectary for tick exposure. Larvae were allowed to feed to repletion and molt. **c** Treatment group mice were presented with bait stations loaded with fipronil bait. **d** Mice transferred to the insectary for tick exposure. Fipronil bait prevents larvae from feeding to repletion and molting

The primary objective of the current study was to investigate the efficacy of a low-dose fipronil bait, presented to white-footed mice, in controlling blood-feeding blacklegged tick larvae under simulated field conditions conducted in the laboratory. Mice were group-housed in large enclosures and offered fipronil bait and an alternative diet in a choice test. Fipronil bait was presented in a commercial bait station. Groups of mice were assigned to 24-h (reduced exposure) or 168-h (extended exposure) fipronil bait exposure durations. For mice receiving reduced exposure, ticks were allowed to attach and blood-feed at days 1 and 15 post-exposure. For mice receiving extended exposure, ticks were allowed to attach and feed at days 21 and 35 post-exposure. Ticks were observed via microscopy and were collected once fed to repletion and significant differences between treatment and control groups were estimated. A conceptual diagram describing the purpose of the experiment is presented in Fig. 1. The results of this study should provide additional insights regarding the use of a potential new control tool that could be integrated into tick management programs.

### White-footed mice and blacklegged ticks

White-footed mice utilized in testing were from a previously described outbred breeding colony [17]. Larvae were acquired from the Oklahoma State Tick Rearing Facility (Stillwater, OK, USA) and maintained in a regulated insectary under previously specified conditions [17].

All procedures performed during this study involving white-footed mice, and the test protocol, were approved by the Genesis Institutional Animal Care and Use Committee (IACUC) (August 6, 2020) and followed Animal Welfare Act (AWA) and Genesis IACUC policies (Study No. 20003).

### Fipronil bait

The manufactured bait (Scimetrics, Inc., Wellington, CO, USA) was the same formulation used during our previous experiment [17]. The nominal fipronil concentration was 0.005% (50 mg/kg) and the mean fipronil concentration was confirmed to be 46.3 ± 1.32 mg/kg (CV: 2.86%; Recovery: 92.6%) using previously described high-performance liquid chromatography (HPLC) methods [17], which is within the requirements outlined by the US Environmental Protection Agency (EPA) (±10%).

### Experimental design

#### Pre-exposure (acclimation)

During acclimation, mice were housed in groups of 10, separated by sex, in metal stock tanks (enclosures) having a surface area ~ 46,700 cm^2^. Wood shavings were used to absorb urine and feces and were replaced weekly. Enclosures were each equipped with a single animal shelter and cotton to simulate bedding material.

Mice were acclimated to test conditions for a minimum of 3 days. Environmental conditions in test rooms (temperature, relative humidity) and the general health of test mice were monitored daily. All mice were provided commercial rodent diet (Envigo, Indianapolis, IN, USA) and tap water (via glass bottle) ad libitum. An attending veterinarian inspected all animals prior to exposure to ensure study suitability.

#### Exposure: group assignment

Mice were assigned to groups using a random sequence generator. Mice were separated into groups differentiated based on (1) test group identity (treatment, control) and (2) the length of the bait exposure (24 h, 168 h). A total of 40 mice (20 male, 20 female) underwent a bait exposure period of 24 h (reduced). An additional 40 mice (20 male, 20 female) underwent a 168-h bait exposure period (extended). The reduced exposure length was selected to determine deliverability of fipronil bait to group-housed mice over 24 h and to determine whether neophobia would be an issue initially [22]. Extended exposure was selected to determine whether fipronil could be safely delivered to group-housed mice for a minimum of 1 week. Mice continued to be housed in the metal stock tanks during exposure and were separated by (1) sex and (2) test group ID. The enclosure specifics are listed in Table 1.

**Table 1:** Summary of the mouse enclosures utilized during acclimation, exposure, and post-exposure

Enclosure ID	Sex	Test group ID	Treatment/control	Bait exposure duration	No. mice
1	Male	T24	Treatment	24 h (reduced)	10
2	Female	T24	Treatment	24 h (reduced)	10
3	Male	C24	Control	24 h (reduced)	10
4	Female	C24	Control	24 h (reduced)	10
5	Male	T168	Treatment	168 h (extended)	10
6	Female	T168	Treatment	168 h (extended)	10
7	Male	C168	Control	168 h (extended)	10
8	Female	C168	Control	168 h (extended)	10

#### Exposure

**Fig. 2:**
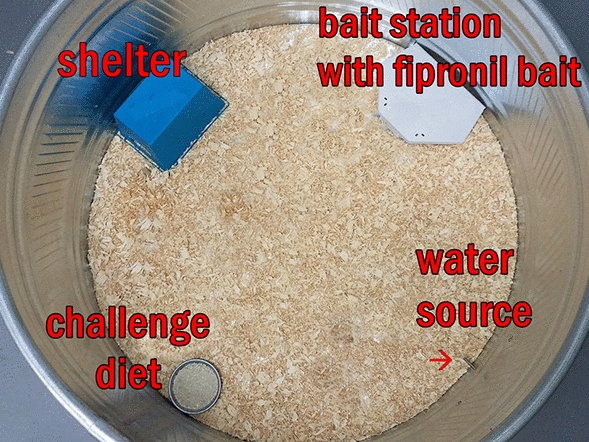
Overhead view of an enclosure housing 10 treatment group mice. The bait station and alternative diet are positioned equidistance from the water source and shelter

At the end of acclimation, all commercial rodent diet was removed from the enclosures. At initiation of the exposure period, each treatment group enclosure (10 mice) received approximately 100 g fipronil bait presented in a single commercial bait station (Protecta^®^ LP, Bell Laboratories, Inc., Windsor, WI, USA) and approximately 100 g of US EPA field rodent challenge diet (CD) [23] in an open food container. CD is a mixture of commercial rodent diet and rolled oats (50:50 ratio) recommended by the US EPA for use in choice tests involving *Peromyscus* spp. [23]. The fipronil bait station and CD were positioned against the wall of the enclosure at opposing sides and were positioned equidistant from the water source and shelter (Fig. 2). Each control group was presented CD exclusively in two open containers.

#### Reduced exposure

Fipronil bait was presented to white-footed mice within the treatment groups for 24 h using the previously described methodology. At the conclusion of the 24-h exposure, all fipronil bait and CD were removed and replaced with commercial rodent diet. The remaining fipronil bait and challenge diet were weighed to the nearest 0.1 g.

#### Extended exposure

Fipronil bait was presented to white-footed mice within the treatment groups for 168 h using the previously described methodology. The fipronil bait and CD were removed once every 24 h, weighed to the nearest 0.1 g, and immediately returned to the enclosures. Fipronil bait and CD were replenished ad libitum. At the conclusion of 168 h, all fipronil bait and challenge diet were removed and replaced with commercial rodent diet.

At the conclusion of exposure (reduced, extended), all bedding was removed and replaced with clean bedding to ensure that no fipronil bait was present in the enclosures.

#### Post-exposure

During post-exposure, mice remained in the group enclosures and were provided commercial rodent diet and tap water ad libitum and were observed daily for general health. Mice remained in group enclosures until tick attachment.

#### Tick attachment: subgroup assignment

Mice were further separated into subgroups differentiated based on the time point of tick attachment. Mice within each test group were randomly assigned to subgroups using a random sequence generator. Ticks were inserted into capsules attached to 10 mice (5 male, 5 female) within each subgroup. EPA guidelines recommend a sample size of 10 subjects per group, with a minimum allowance of 6 when evaluating pesticides against pests of humans and pets such as fleas and ticks [24]. Additionally, a power analysis was conducted to determine the recommended sample size using an analysis of variance (G*Power 3.1.9.2, University of Kiel, Germany). An effect size of 0.8 was selected based on the ≥ 80% removal of ticks required by the EPA to make “controls ticks” claims [24]. An error probability of 0.05 and power of 0.90 were selected. The results of the power analysis suggested a minimum total sample size of 40 (5/group) (actual power = 0.936916). This suggested the use of 6–10 mice per test group to be acceptable.

A summary of the groups and subgroups utilized can be seen in Table 2.

**Table 2:** Study specifics for each group and subgroup of white-footed mice utilized

Bait exposure duration	Test group ID	Subgroup ID	Day of tick application (post-bait exposure)	No. mice	No. larvae/mouse
Reduced	T24	T24-1	Day 1	10	40
		T24-15	Day 15	10	40
	C24	C24-1	Day 1	10	40
		C24-15	Day 15	10	40
Extended	T168	T168-21	Day 21	10	40
		T168-35	Day 35	10	40
	C168	C168-21	Day 21	10	40
		C168-35	Day 35	10	40

#### Tick attachment

At the initiation of each tick attachment period, all treatment and control group mice within the appropriate subgroups were transferred from the animal study room to the insectary where they were housed in individual wire cages each suspended above a moat of water used to collect detached larvae. Forty larval ticks were applied to each mouse within a small capsule, which is a preferred method for localizing blood-feeding for blacklegged tick larvae infesting mice [25–28]. All tick and animal procedures utilized in tick attachment are explicitly described in our previous work [17].

#### Post-tick attachment

**Fig. 3:**
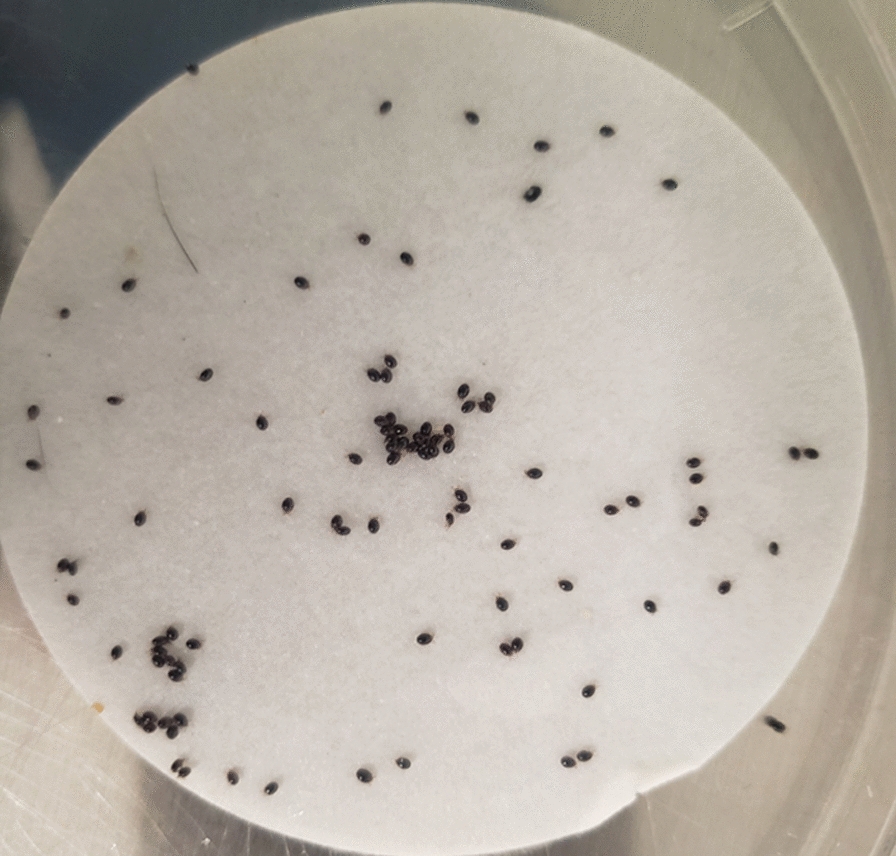
Replete blacklegged tick larvae collected from moats being dried on a sheet of filter paper

**Fig. 4:**
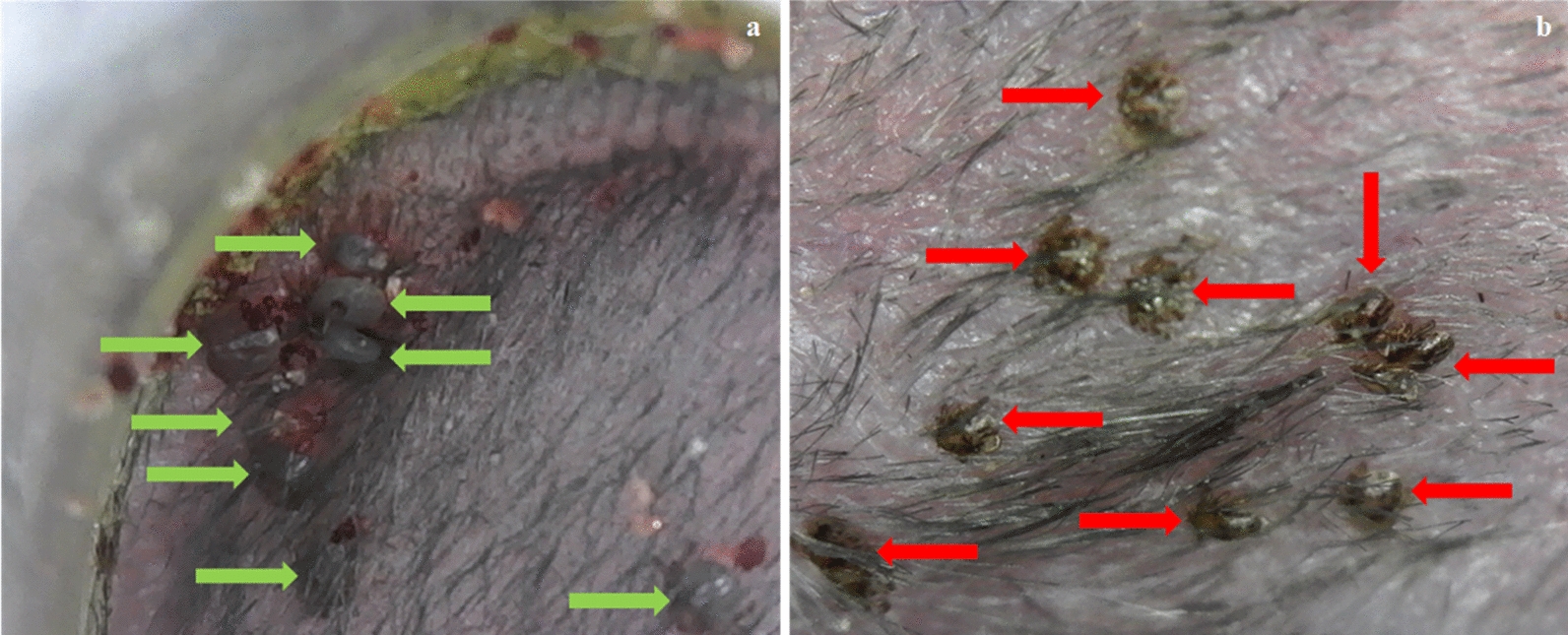
Comparison of **a** engorging larvae feeding on control mouse, and **b** non-engorged, deceased larvae on treatment mouse. Engorging larvae are bloated and excreting red feces (green arrows). Non-engorged, deceased larvae are brown and desiccated (red arrows)

**Fig. 5:**
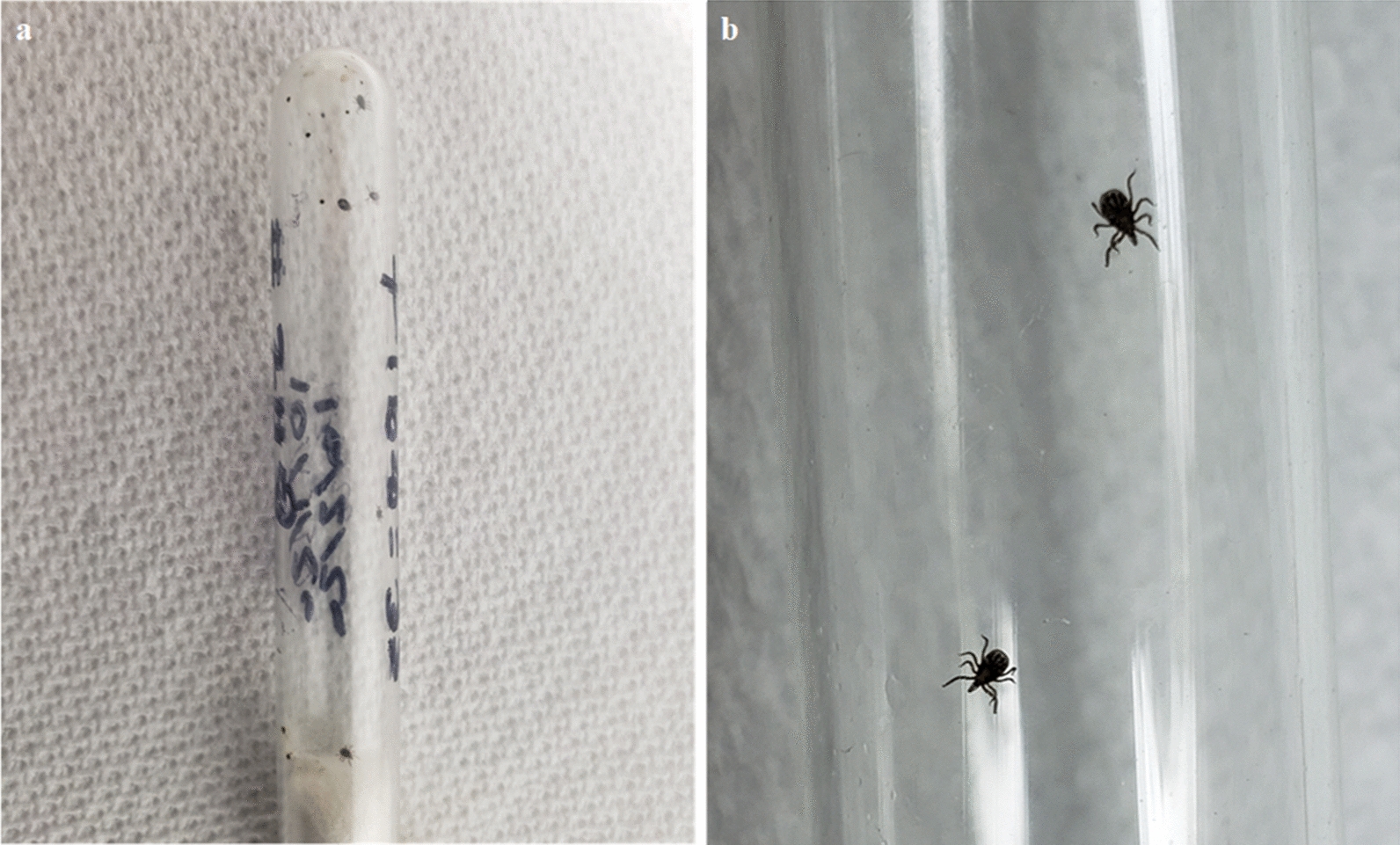
**a** Test tube containing replete larvae and molted nymphs, **b** closer view of a test tube with several molted nymphs

The post-tick attachment procedures are explicitly described in our previous work [17]. During the tick feeding period (post-tick attachment), three methods of observing and recovering ticks were used: (i) collecting non-engorged and replete ticks from the water in moats, (ii) observing attached ticks within the capsules through microscopy, and (iii) monitoring detached replete larvae for molting.

i. *Moat observations and tick recovery* Twice daily, the water in the moats under each cage was searched for non-engorged or replete ticks (Fig. 3) in the same manner as described previously [17].
ii. *Microscope tick observations* The inside of each capsule was carefully scanned for attached non-engorged larvae (brown, often desiccated) and engorging larvae (bloated and white, gray, red or pink in color, generally surrounded by red feces) (Fig. 4) in the same manner as described previously [17].
iii. *Monitoring of detached replete larvae* Post-repletion represents the 8-week (56-day) period immediately following the final day of each post-tick attachment period. During this time, replete larvae within the control subgroups and treatment subgroups were retained separately in glass test tubes (Fig. 5). Dense cotton was placed into the opening of the tube to prevent escape of larvae while allowing adequate air exchange. At the conclusion of the post-repletion period, each test tube was carefully scanned for molted nymphs. To do this, each tube was divided into quadrants of equal size. The tube was placed into a cooler filled with icepacks for ~ 5 min to slow tick movement. Molted nymphs and replete larvae were then counted within each tube.

A schedule detailing the dates of acclimation, exposure, post-exposure, and post-tick attachment for each test group is presented in Additional file 1.

#### Blood sample collection

At the conclusion of day 4 post-tick attachment, blood samples were taken from treatment subgroup mice. Unlike our previous study [17], where blood was taken from four randomly selected mice from each group, blood samples were collected from all treatment group mice in this study. This decision was made because mice were group housed, making bait consumption from individual mice unattainable. Thus, the fipronil concentration in plasma (CP) for individual plasma samples would provide the only indication that individual mice had consumed the bait. Blood samples were collected from four control group mice to establish a baseline.

Mice were first anesthetized and euthanized in accordance with AWA recommended procedures [29], and approximately 100 µl of blood was collected from each animal. Blood samples were then spun in a centrifuge after which plasma was delivered to the Center for Environmental Medicine (CEM) Analytical Laboratory at Colorado State University (Fort Collins, CO, USA) for analysis of CP. These methods are explicitly described in our previous work [17].

### Data analyses

#### White-footed mouse body weight

The body weight of all mice was recorded prior to fipronil bait exposure and at the conclusion of the post-tick attachment period. Differences in body weight between test groups (treatment vs. control) and within each test group (initial weight vs. final weight) were estimated using a Wilcoxon signed-rank test, where *p* ≤ 0.05 was considered significant [30].

#### Fipronil bait consumption

Fipronil bait consumption was estimated daily (to the nearest 0.1 g) for each mouse group. We then used the total bait consumed in each group to estimate the average total fipronil consumed by each mouse each day. The body weights taken prior to fipronil bait exposure were used to estimate total fipronil consumption in mg/kg per individual mouse.

#### Tick observations and recovery

All larvae collected from moats, observed via microscopy, and monitored for molting were explicitly defined based on developmental status. The specific definitions are listed below:

##### Moats

Non-engorged = Flat larvae, showing no discernable blood meal, collected from moats.

Replete = Fully engorged, darkly colored larvae collected from moats.

##### Microscopy

Non-engorged = Attached larvae, expired and desiccated and/or having no discernable blood meal. Observed within the capsule via microscopy.

Engorging = Attached, actively feeding, bloated larvae observed in the capsule via microscopy.

##### Post-repletion

Replete = Engorged, darkly colored larvae in test tube.

Molted = Fully developed nymph.

Differences in (i) body weight, (ii) the numbers of non-engorged and replete larvae collected from moats per test group, (iii) attached non-engorged and engorging larvae within capsules per test group, and (iv) larvae within capsules successfully detaching per test group were analyzed (Wilcoxon signed-rank test: *p* ≤ 0.05).

#### Fipronil plasma concentration

The CP (ng/ml) was estimated for each individual mouse euthanized (*n* = 43). The limit of quantification (LOQ) was 1.25 ng fipronil/ml plasma. Comparisons were made between the numbers of replete and non-engorged larvae collected from each mouse, relative to the presence of fipronil in plasma samples (Wilcoxon signed-rank test: *p* ≤ 0.05).

#### Mortality estimates

##### Preventing attached larvae from feeding to repletion

The efficacy of fipronil bait in preventing attached blacklegged tick larvae from feeding to repletion was calculated using Abbott’s formula [31] to account for the control groups.

$${\text{Efficacy}}(\% ) = 100*\left( {\frac{{{\text{Ca}} - {\text{Ta}}}}{{{\text{Ca}}}}} \right),$$

where Ca = mean number of attached larvae feeding to repletion per mouse in the control subgroup; Ta = mean number of attached larvae feeding to repletion per mouse in the treatment subgroup.

##### Preventing detachment of larvae observable within the capsule by microscope

The post-tick attachment microscope observations were used to estimate the number of attached larvae successfully detaching from the host. The total numbers of attached larvae observable within the capsules successfully detaching from each mouse was estimated using the following formula:

*Total successfully detached* = *Total attached (Day 2)* − *Total attached (Day 4).*

*Total attached* = non-engorged + engorging. If *Total attached (Day 4)* was equal to or greater than *Total attached (Day 2)*, then *Total successfully detached* = 0.

The efficacy of the fipronil bait in preventing blacklegged tick larvae observable within the capsule from successfully detaching was calculated using the Henderson and Tilton formula [32], a modified form of Abbott’s formula:

$${\text{Efficacy}}(\% ) = 100*\left( {1 - \frac{{{{Ta}}*{{Cb}}}}{{{{Tb}}*{{Ca}}}}} \right),$$

where *T *= treatment subgroup, *C *= control subgroup, *b *= total attached (day 2), *a *= total successfully detached.

#### Preventing development to nymphs (molting)

The efficacy of the fipronil bait in preventing replete larvae from molting was calculated using Abbot’s formula, but the variables were redefined as follows:

Ca = mean number of replete larvae molting to nymphs in the control subgroup; Ta = mean number of replete larvae molting to nymphs in the treatment subgroup

All statistics and data analyses were performed using the current versions of JMP Statistical Software (Version 15) (Cary, NC, USA) and Microsoft Excel.

## Results

### Mouse body weight

A summary of body weight values can be found in Table 3. Within the treatment groups, average initial body weights were 22.1 g (reduced) and 21.2 g (extended), and the average final body weights were 22.3 (reduced) and 20.7 (extended). Within the control groups, average initial body weights were 21.8 g (reduced) and 21.6 g (extended) and the final body weights were 21.5 g (reduced) and 21.4 g (extended). All mice proceeding to the exposure and post-exposure periods had initial weight within the EPA recommended allowances (15–40 g) [23]. No significant differences were detected between treatment and control when comparing initial weights within the reduced (Wilcoxon signed-rank test: *Z* = 0.2571, *p* = 0.7971) and extended (*Z* = −0.23891, *p* = 0.8112) test groups or final weights in the reduced (*Z* = 0.9472, *p* = 0.3435) and extended (*Z* = −0.3092, *p* = 0.7572) test groups. Final body weight did not differ significantly from initial weight within the reduced (*Z* = −0.2707, *p* = 0.7866) or extended (*Z* = 0.7012, *p* = 0.4832) treatment groups or the reduced (*Z* = 0.4194, *p* = 0.6749) or extended (*Z* = 0.2165, *p* = 0.8286) control groups.

**Table 3:** Initial and final body weight (g) for white-footed mice within each test group (mean ± SD)

Test group	Bait exposure	Sex	Body weight (g)	
			Initial	Final
Treatment	Reduced	Male	22.3 ± 2.0	22.7 ± 1.8
		Female	21.9 ± 3.1	21.8 ± 2.4
		Total	22.1 ± 2.6	22.3 ± 2.1
	Extended	Male	21.2 ± 1.9	21.5 ± 2.0
		Female	20.9 ± 1.9	19.9 ± 2.5
		Total	21.2 ± 1.9	20.7 ± 2.3
Control	Reduced	Male	23.0 ± 2.6	22.5 ± 1.9
		Female	20.6 ± 2.0	20.4 ± 2.0
		Total	21.8 ± 2.2	21.5 ± 2.2
	Extended	Male	21.8 ± 1.9	22.4 ± 3.1
		Female	21.3 ± 4.6	20.4 ± 2.7
		Total	21.6 ± 3.5	21.4 ± 3.0

### Feed consumption

Results indicated that fipronil bait was palatable in the presence of CD feed (Table 4). On average, mice within each treatment group consumed more fipronil bait, relative to CD. In the reduced exposure group, fipronil bait made up 89.8% and 82.9% of the total diet consumed by male and female mice, respectively. In the extended exposure group, fipronil bait made up 53.1% and 74.0% of the total diet for male and female mice, respectively.

**Table 4:** Comparison of consumption of fipronil bait and challenge diet (CD) within the treatment and control groups of white-footed mice

Treatment				Control			
Test group	Sex	Diet	Consumption (g)	Test group	Sex	Diet	Consumption (g)
Reduced bait exposure	Male (*n* = 10)	Fipronil bait	38.0	Reduced bait exposure	Male (*n* = 10)	CD	42.0
		CD	4.3				
	Female (*n* = 10)	Fipronil bait	31.6		Female (*n* = 10)	CD	42.7
		CD	6.5				
Extended bait exposure	Male (*n* = 10)	Fipronil Bait	138.4	Extended bait exposure	Male (*n* = 10)	CD	273.2
		CD	122.0				
	Female (*n* = 10)	Fipronil bait	136.3		Female (*n* = 10)	CD	170.8
		CD	48.0				

A summary of the average fipronil consumed (mg/kg) per mouse is presented in Table 5. Male mice within the reduced treatment group consumed an average of 8.5 mg fipronil/kg body weight. Female mice within the reduced treatment groups consumed an average of 7.3 mg/kg. Mice within extended treatment groups consumed an average of 4.7 mg/kg/day (male) and 4.8 mg/kg/day (female).

**Table 5:** Estimated individual fipronil consumption by treatment group white-footed mice (*n* = 40)

Treatment group	Sex	Mean body weight ± SD (g)	Mean total bait consumed/mouse (g)	Mean bait consumed/mouse/day (g)	Mean daily fipronil consumed/mouse (mg)	Mean daily fipronil consumed/mouse (mg/kg)
Reduced bait exposure	Male	22.3 ± 2.0	3.8	3.8	0.19	8.5
	Female	21.9 ± 3.1	3.2	3.2	0.16	7.3
Extended bait exposure	Male	21.2 ± 1.9	13.8	2.0	0.10	4.7
	Female	20.9 ± 1.9	13.6	1.9	0.10	4.8

The amount of fipronil bait eaten by treatment group females (reduced and extended) was significantly greater than for CD feed (Wilcoxon signed-rank test: *Z* = 3.3106, *p* = 0.0009). Males within the treatment groups consumed more fipronil bait than CD feed, but the differences were not determined to be significant (*Z* = 1.2077, *p* = 0.2271). The consumption exceeded requirements of test substance acceptance (33%) outlined in EPA anticoagulant rodenticide protocols [23].

### Mouse observations

Seventy-nine of 80 mice appeared normal and healthy throughout the study. One female mouse was found dead within the extended exposure group (T168-35). However, no signs of toxicity were observed. It is suspected that fighting caused the death.

### Tick observations and recovery

In total, 3160 blacklegged tick larvae were introduced onto 79 test group mice (control = 1600, treatment = 1560).

#### Collection moats

In the control groups and treatment groups, totals of 371 (23.2% of total larvae introduced) and 464 (29.7% of total larvae introduced) non-engorged larvae were respectively collected from within moats (Table 6). Most non-engorged larvae were collected during the early stages of post-tick attachment, with 321 (86.5%) within the control groups being collected at or before day 1 post-tick attachment and 325 (70%) within the treatment groups collected at or before day 1. Non-engorged larvae were collected from the collection tubs of every single test mouse. From days 2 to 4, the number of non-engorged larvae collected within the treatment groups (*n* = 139) was noticeably larger relative to that in the control groups (*n* = 50).

**Table 6:** Summary of non-engorged and replete blacklegged tick larvae collected from moats per day post-tick attachment

Mouse test group	Larvae	Number of larvae collected from moats (days post-tick attachment)									
		Day 1		Day 2		Day 3		Day 4		Total	
		Non-engorged	Replete	Non-engorged	Replete	Non-engorged	Replete	Non-engorged	Replete	Non-engorged	Replete
C24-1	400	53	0	2	0	2	40	1	120	58	160
T24-1	400	51	0	10	0	10	0	5	0	76	0
C24-15	400	61	0	12	0	4	13	1	105	78	118
T24-15	400	82	0	17	0	13	2	6	11	118	13
C168-21	400	91	0	9	0	7	12	0	129	107	141
T168-21	400	80	0	26	0	15	1	6	19	127	20
C168-35	400	116	0	11	0	1	3	0	106	128	109
T168-35	360	112	0	20	0	11	5	0	19	143	24
Control total	1600	321	0	34	0	14	68	2	460	371	528
Treatment total	1560	325	0	73	0	49	8	17	49	464	57

Within the treatment subgroups a total of 57 detached replete larvae (3.7% of total larvae introduced) were collected over the course of all post-tick attachment periods (Tables 6, 7). Replete ticks collected from T24-1, T24-15, T168-21, and T168-35 totaled 0, 13, 20 and 24, respectively. Within the treatment subgroups, the proportion of mice from which replete larvae were collected was 0% (T24-1), 10.0% (T24-15), 40.0% (T168-21), and 88.9% (T168-35). Within the control subgroups, a total of 528 detached replete larvae (33.0% of total larvae introduced) were collected over the course of all post-tick attachment periods (Tables 6, 7). Replete ticks collected from C24-1, C24-15, C168-21, and C168-35 totaled 160, 118, 141, and 109, respectively. All mice within all control subgroups had replete larvae collected from them, at days 3 and 4 post-tick attachment.

**Table 7:** Summary of the total number and mean non-engorged and replete blacklegged tick larvae collected from moats over the course of post-tick attachment

Mouse test group	Total larvae introduced onto mice	Larvae collected from moats					
		Total non-engorged larvae recovered	Mean ± SD non-engorged larvae per mouse	Proportion of mice with non-engorged larvae (%)	Total replete larvae recovered	Mean ± SD replete larvae per mouse	Proportion of mice with replete larvae (%)
C24-1	400	58	5.8 ± 4.5	100	160	16.0 ± 4.9	100
T24-1	400	76	7.6 ± 4.6	100	0	0	0
C24-15	400	78	7.8 ± 2.9	100	118	11.8 ± 3.9	100
T24-15	400	118	11.8 ± 5.6	100	13	1.3 ± 4.1	10
C168-21	400	107	10.7 ± 4.2	100	141	14.1 ± 2.8	100
T168-21	400	127	12.7 ± 6.7	100	20	2.0 ± 3.3	40
C168-35	400	128	12.8 ± 5.8	100	109	10.9 ± 2.6	100
T168-35	360	143	15.9 ± 7.0	100	24	2.7 ± 1.7	88.9
Control total	1600	371	9.3 ± 5.1	100	528	13.2 ± 4.1	100
Treatment total	1560	464	11.9 ± 6.5	100	57	1.5 ± 2.9	33.3

The control subgroups had a significantly greater number of replete ticks collected, relative to treatment, when comparing the following subgroups: T24-1 vs. C24-1 (Wilcoxon signed-rank test: *Z* = −5.2792, *p* < 0.0001), T24-15 vs. C24-15 (*Z* = −3.8491, *p* < 0.0001), T168-21 vs. C168-21 (*Z* = −2.9884, *p* = 0.0028). No significant differences were detected when making similar comparisons for non-engorged ticks collected from the moats.

#### Capsule observations

A summary of capsule observations is presented in Table 8. Cumulatively, within the control subgroups, the number of observable non-engorged larvae decreased from a total of 104 at day 2 to 19 observed at day 4. Moreover, within the control subgroups, the number of engorging larvae decreased from 363 at day 2 to 93 at day 4, which was reflected in the number of replete larvae that were collected in moats. Contrarily, within the treatment subgroups, the number of observable non-engorged larvae increased from 423 at day 2 to 463 at day 4, and the number of engorging larvae deceased from 60 at day 2 to 25 at day 4.

**Table 8:** Mean number of attached blacklegged tick larvae (±SD) per mouse observable within each capsule within each test group

Test group	Observable attached ticks, post-tick attachment							
	Day 2				Day 4			
	Non-engorged		Engorging		Non-engorged		Engorging	
	Total	Mean ± SD	Total	Mean ± SD	Total	Mean ± SD	Total	Mean ± SD
C24-1	16	1.6 ± 2.0	107	10.7 ± 2.5	1	0.1 ± 0.3	14	1.4 ± 1.6
T24-1	125	12.5 ± 2.1	0	0	132	13.2 ± 2.3	0	0
C24-15	33	3.3 ± 1.3	82	8.2 ± 2.7	3	0.3 ± 0.5	21	2.1 ± 1.0
T24-15	136	13.6 ± 2.5	7	0.7 ± 1.3	147	14.7 ± 2.9	4	0.4 ± 1.0
C168-21	25	2.5 ± 1.8	95	9.5 ± 4.0	6	0.6 ± 1.0	26	2.6 ± 2.0
T168-21	107	10.7 ± 2.4	16	1.6 ± 2.3	114	11.4 ± 2.3	4	0.4 ± 0.7
C168-35	30	3.0 ± 2.4	79	7.9 ± 2.0	9	0.9 ± 1.1	32	3.2 ± 1.6
T168-35	55	6.1 ± 2.7	37	4.1 ± 1.4	70	7.8 ± 2.6	17	1.9 ± 1.4
Control total	104	2.6 ± 2.0	363	9.1 ± 3.0	19	0.5 ± 0.8	93	2.3 ± 1.7
Treatment total	423	10.8 ± 3.7	60	1.5 ± 2.1	463	11.9 ± 3.5	25	0.6 ± 1.1

The control subgroups had a significantly greater number of engorging larvae observable during post-tick attachment, relative to treatment, when comparing the following subgroups: T24-1 vs. C24-1 (Wilcoxon signed-rank test: *Z* = −6.3702, *p* < 0.0001), T24-15 vs. C24-15 (*Z* = −6.1035, *p* < 0.0001), T168-21 vs. C168-21 (*Z* = −5.5535, *p* < 0.0001), and T168-35 vs. C168-35 (*Z* = −4.3842, *p* < 0.0001). The treatment subgroups had a significantly greater number of larvae that were non-engorged, relative to control, when comparing the following groups: T24-1 vs. C24-1 (*Z* = 6.7885, *p* < 0.0001), T24-15 vs. C24-15 (*Z* = 6.6714, *p* < 0.0001), T168-21 vs. C168-21 (*Z* = 6.6876, *p* < 0.0001), and T168-35 vs. C168-35 (Z = 5.8412, *p* < 0.0001).

#### Nymphal development (molting)

A summary of molting success is presented in Table 9. Molting was not monitored for the T24-1 and C24-1 subgroups because no replete larvae were collected from T24-1 mice. Of the 13 replete larvae collected within T24-15 (all from a single mouse), 7 (53.8%) molted by the end of the post-repletion period. Of the 118 larvae collected within C24-15, 79 (66.9%) molted. Of the replete larvae collect from T168-21 (20) and C168-21 (141) a total of 8 (40%) and 96 (68.1%) molted, respectively. Of the replete larvae collect from T168-35 (24) and C168-35 (109) a total of 13 (54.2%) and 80 (73.4%) molted, respectively. Within the treatment groups, the proportions of mice which had larvae successfully molt were 0% (T24-1), 10% (T24-15), 30% (T168-21), and 66.7%.

**Table 9:** Summary of replete blacklegged tick larvae successfully molting for each test group

Test group ID	Total mice	Mean larvae placed in each capsule day 0	Total replete larvae placed in desiccator	Mean replete larvae placed in desiccator	Total replete larvae molting	Mean replete larvae molting
C24-15	10	40	118	11.8	79	7.9
T24-15	10	40	13	1.3	7	0.7
C168-21	10	40	141	14.1	96	9.6
T168-21	10	40	20	2	8	0.8
C168-35	10	40	109	10.9	80	8
T168-35	9	40	24	2.7	13	1.4

### Fipronil plasma concentration

A list of all plasma samples analyzed for CP is presented in Table 10. Fipronil sulfone was the only metabolite detectable > LOQ of 1.25 ng/ml. All mice within the T24-1 group had CP at levels detectable > LOQ. Within the T24-15 group, 9 of 10 mice had CP detectable > LOQ, and all replete ticks (*n* = 13) were collected from the mouse that had no CP detectable > LOQ. Within the T168-21 group, 6 of 10 mice had CP detectable > LOQ. Within T168-35, only 1 of 9 mice had CP detectable > LOQ, and this single mouse was the only one which had no replete ticks collected from within T168-35. Moreover, a single replete larva was collected off a single mouse (Mouse #113) from T168-21 with CP detectable > LOQ (4.8 ng/ml), but the larva did not molt. The mean CP per mouse was 191.5 ng/ml (T24-1), 29.4 ng/ml (T24-15), 10.6 ng/ml (T168-21), and 1.0 ng/ml (T168-35), and 0 (control).

**Table 10:** Fipronil sulfone concentrations in white-footed mice utilized in tick attachments

Sex	Group ID	Group	Exposure	Attachment time point (post-exposure)	Blood collection time point (post-exposure)	Fipronil sulfone (ng/ml)	Number of replete ticks successfully detaching
Male	T24-1	Treatment	24 h	Day 1	Day 5	96.3	0
Male	T24-1	Treatment	24 h	Day 1	Day 5	140.3	0
Male	T24-1	Treatment	24 h	Day 1	Day 5	222.7	0
Male	T24-1	Treatment	24 h	Day 1	Day 5	247.4	0
Male	T24-1	Treatment	24 h	Day 1	Day 5	361.9	0
Female	T24-1	Treatment	24 h	Day 1	Day 5	161.5	0
Female	T24-1	Treatment	24 h	Day 1	Day 5	234.7	0
Female	T24-1	Treatment	24 h	Day 1	Day 5	75.6	0
Female	T24-1	Treatment	24 h	Day 1	Day 5	134.4	0
Female	T24-1	Treatment	24 h	Day 1	Day 5	239.7	0
Male	T24-15	Treatment	24 h	Day 15	Day 19	78.8	0
Male	T24-15	Treatment	24 h	Day 15	Day 19	15.5	0
Male	T24-15	Treatment	24 h	Day 15	Day 19	ND	13
Male	T24-15	Treatment	24 h	Day 15	Day 19	12.7	0
Male	T24-15	Treatment	24 h	Day 15	Day 19	40.3	0
Female	T24-15	Treatment	24 h	Day 15	Day 19	18.8	0
Female	T24-15	Treatment	24 h	Day 15	Day 19	16.2	0
Female	T24-15	Treatment	24 h	Day 15	Day 19	4.4	0
Female	T24-15	Treatment	24 h	Day 15	Day 19	79.3	0
Female	T24-15	Treatment	24 h	Day 15	Day 19	28.2	0
Male	T168-21	Treatment	168 h	Day 21	Day 25	32	0
Male	T168-21	Treatment	168 h	Day 21	Day 25	11.9	0
Male	T168-21	Treatment	168 h	Day 21	Day 25	25.7	0
Male	T168-21	Treatment	168 h	Day 21	Day 25	19.4	0
Male	T168-21	Treatment	168 h	Day 21	Day 25	ND	8
Female	T168-21	Treatment	168 h	Day 21	Day 25	ND	0
Female	T168-21	Treatment	168 h	Day 21	Day 25	4.8	1
Female	T168-21	Treatment	168 h	Day 21	Day 25	ND	9
Female	T168-21	Treatment	168 h	Day 21	Day 25	ND	2
Female	T168-21	Treatment	168 h	Day 21	Day 25	11.8	0
Male	T168-35	Treatment	168 h	Day 35	Day 39	ND	2
Male	T168-35	Treatment	168 h	Day 35	Day 39	8.8	0
Male	T168-35	Treatment	168 h	Day 35	Day 39	ND	1
Male	T168-35	Treatment	168 h	Day 35	Day 39	ND	5
Male	T168-35	Treatment	168 h	Day 35	Day 39	ND	2
Female	T168-35	Treatment	168 h	Day 35	Day 39	ND	5
Female	T168-35	Treatment	168 h	Day 35	Day 39	ND	2
Female	T168-35	Treatment	168 h	Day 35	Day 39	ND	4
Female	T168-35	Treatment	168 h	Day 35	Day 39	ND	3
Male	C24-1	Control	NA	Day 1	Day 5	ND	19
Female	C24-1	Control	NA	Day 1	Day 5	ND	10
Male	C168-35	Control	NA	Day 35	Day 39	ND	11
Female	C168-35	Control	NA	Day 35	Day 39	ND	7

ND = No fipronil sulfone detected at levels > 1.25 ng/ml (LOQ)

Within the treatment groups, plasma samples with CP detectable > LOQ came from mice with significantly lower numbers of replete larvae collected, relative to samples < LOQ (*Z* = 5.4908, *p* < 0.0001). Contrarily, the numbers of non-engorged larvae collected from mice were not significantly different when comparing samples with CP detectable > LOQ with those which did not within the treatment groups (*Z* = 1.1193, *p* = 0.2630).

### Efficacy estimates

#### Reduced bait exposure

The mean number of replete ticks collected per mouse was 0 (T24-1) and 1.3 (T24-15) within the treatment subgroups and 16.0 (C24-1) and 11.8 (C24-15) within the control subgroups. The efficacy of fipronil bait in preventing blacklegged tick larvae from feeding to repletion was estimated to be 100% (T24-1) and 89.0% (T24-15). One hundred percent (100%) efficacy was obtained if the single mouse which yielded replete larvae in the T24-15 group was excluded from analysis.

The mean number of attached larvae, observable within each capsule (engorged + non-engorged), observed at day 2 post-tick attachment was 12.5 (T24-1) and 14.3 (T24-15) within the treatment subgroups and 12.3 (C24-1) and 11.5 (C24-15) within the control subgroups. The mean number of larvae, observable within each capsule, successfully detaching (*Day 2 attached* − *Day 4 attached*) was 0 (T24-1, T24-15) within the treatment subgroups and 10.8 (C24-1) and 9.1 (C24-15) within the control groups. The efficacy of the fipronil bait in preventing blacklegged tick larvae observable within the capsules from detaching was estimated to be 100%. The efficacy estimated for T24-15 conflicts with the repletion efficacy estimated for this subgroup. This is largely due to the following: (1) several larvae that were initially defined as “engorging” at day 2 post-tick attachment eventually succumbed to fipronil bait and thus being redefined as “non-engorging” at day 4, and (2) larvae failing to attach by day 2 being observed attached at day 4.

No replete larvae were collected within T24-1, and thus 100% of ticks on mice were prevented from molting. The mean number of molted nymphs recorded per mouse at the conclusion of the post-repletion period was 1.3 within T24-15 and 7.9 within C24-15. In T24-15, the efficacy of reduced exposure in preventing all ticks on the mice from eventually molting was 91.1%.

#### Extended bait exposure

The mean number of replete ticks collected per mouse was 2.0 (T168-21) and 2.7 (T168-35) within the treatment subgroups and 14.1 (C168-21) and 10.9 (C168-35) within the control subgroups. The efficacy of fipronil bait in preventing blacklegged tick larvae from feeding to repletion was estimated to be 85.8% (T168-21) and 75.2% (T168-35).

The mean number of attached larvae observed at day 2 post-tick attachment was 12.3 (T168-21) and 10.2 (T168-35) within the treatment subgroups 12.0 (C168-21) and 10.9 (C168-35) within the control subgroups. The mean number of larvae, observable within each capsule, successfully detaching was 0.5 (T168-21) and 0.5 (T168-35) within the treatment subgroups and 8.8 (C168-21) and 6.8 (C168-35) within the control subgroups. The efficacy of the fipronil bait in preventing blacklegged tick larvae observable within the capsules from detaching was estimated to be 94.5% (T168-21) and 92.1% (T168-35).

The mean number of molted nymphs recorded per mouse at the conclusion of the post-repletion period was 0.8 (T168-21) and 1.4 (T168-35) within the treatment subgroups and 9.6 (C168-21) and 8.0 (C168-35) within the control subgroups. The efficacy of extended exposure in preventing all ticks on the mice from eventually molting was 91.7% (T168-21) and 82.5% (T168-35).

## Discussion

These results expand upon those from our previous study [17] and provide further insights into the use of a 0.005% fipronil bait in controlling blacklegged ticks parasitizing white-footed mice. The results suggest fipronil bait may be effective at reduced and extended exposure durations and up to 35 days post-fipronil bait exposure. If fipronil bait is to be utilized in integrated tick management programs, the results of the current study should aid in developing appropriate application procedures.

The study indicated that reduced exposure could significantly control larvae up to 15 days post-exposure. During the study, 100% of larvae feeding on mice were prevented from feeding to repletion when attaching at day 1 post-exposure. Additionally, 89.0% of larvae were prevented from feeding to repletion and 91.1% were prevented from molting when attaching at day 15 post-exposure. We should reiterate that all replete larvae and molted nymphs observed at day 15 were recovered from a single mouse, which was the only mouse in this group to have no CP detectable > LOQ. Thus, all mice having CP detectable > LOQ had no replete larvae collected from them. The fact that 19 of 20 mice within the reduced exposure group consumed enough fipronil bait to disrupt feeding by 100% of attached larvae at days 1 and 15 post-exposure indicates that neophobia is not a significant concern for the tested bait formulation.

The results from this study suggest that fipronil bait is palatable even in the presence of an alternative food source. At 24-h exposure, the majority of the mouse diet was fipronil bait for both male (90.7%) and female (82.9%) mice. The alternative diet is a mixture of commercial rodent diet and rolled oats (50:50 ratio) [23] and the rodent diet was the same type used to feed the mice during acclimation. This further dispelled any concerns that mice might demonstrate neophobia during initial bait application. Poché et al. [17] suggested that neophobia may not be of concern because there was no significant difference in the amount of fipronil bait consumed at day 1 and day 2 post-exposure. It should be noted that, in the wild, it is unlikely that rodent density would be as high around bait stations relative to the current study. White-footed mice have an estimated home range size of ~ 2025 m^2^ to ~ 6070 m^2^ and population density is roughly 4 to 12 mice per ~ 4050 m^2^ [33]. Thus, a larger proportion of local mice might consume adequate quantities of bait under field conditions relative to the laboratory because of reduced competition with rival individuals.

The study further indicated the potential for extended exposure to significantly reduce larvae up to 35 days post-exposure. During the study, fipronil bait prevented 85.8% and 75.2% of larvae from reaching repletion at day 21 and day 35 post-exposure, respectively. Further, fipronil bait reduced molting by 91.7% and 82.5% at days 21 and 35 post-exposure, respectively. Mice exposed to fipronil bait for the extended duration showed no observable signs of fipronil toxicity over the 168-h exposure period, suggesting that fipronil bait could be maintained under field conditions for at least 1 week. The ability to keep the bait in the field for extended periods could significantly reduce the labor required when positioning bait stations and would increase the probability of effectively treating a large proportion of white-footed mice in an area.

Mice in the reduced exposure groups consumed fipronil at an average rate of 8.5 (male) and 7.3 (female) mg/kg. Mice in the extended exposure groups consumed fipronil at an average rate of 4.7 (male) and 4.8 (female) mg/kg/day. The increased rate of consumption during the reduced exposure, suggested that the mice experienced a neophiliac response to the initial presence of the bait stations. No symptoms of fipronil toxicity were observed during the experiment, suggesting that fipronil bait could be safely applied under field conditions for extended durations. It is not surprising that no adverse effects were observed in the reduced exposure group, considering the oral LD_50_ of fipronil in mice is approximately 95 mg/kg [34]. The low rate of consumption in the extended exposure groups suggests that fipronil bait may be positioned in the field for extended durations. However, additional research evaluating any potential effects of chronic exposure of fipronil bait to mice could be beneficial. Field studies would be useful in evaluating the ability of commercial bait stations to prevent access to fipronil bait by non-target species. However, it may be advantageous for some additional wildlife species such as chipmunks, which can also serve as *B. burgdorferi* s.s. reservoirs, to have access to the bait as well.

A difference between the results presented here and our previous study [17] is that the former saw significant weight increases within the treatment groups. During the current study, no significant weight changes were observed within any of the test groups. This was likely a byproduct of the mice being housed in large enclosures (~ 46,700 cm^2^) for the majority of the time in the current study, providing more opportunity for movement which can significantly impact weight gain in mice [35, 36]. Poché et al. [17] housed mice in individual 550 m^2^ cages for the entire study duration, and thus mice were afforded less mobility and opportunity for exercise than during the current study. The fact that significant weight loss was not observed in any of the treatment groups provides further indication that the bait can be administered over extended durations.

More non-engorged larvae were observable within the capsules within the treatment groups at day 4 post-tick attachment, relative to day 2, which impacted our estimates of the efficacy of fipronil bait in preventing detachment of larvae observable within the capsule by microscopy (particularly evident in T24-15). The increase at day 4 occurred for two reasons: (1) some larvae defined as “engorging” at day 2 succumbed to fipronil bait and thus were redefined as “non-engorging” larvae at day 4, and (2) larvae not yet attached, or not yet observable within the capsule via microscopy, at day 2 were observed attached at day 4. This was not a significant issue during our previous study [17] because 100% control was obtained within all treatment groups. While capsule observations provide a clear indication of the significant impact fipronil bait has on the feeding and survivorship of attached larvae, our study indicates that repletion success (moat collections) and subsequent molting success provide more appropriate indicators of fipronil bait efficacy. Many researchers have noted difficulties related to blood-feeding and tick recovery from infested animals in experimental settings [17, 27, 37, 38]. Larvae are ≤ 0.8 mm in length, and thus can easily escape notice in containment apparatuses. The current research resulted in slightly greater recovery of larvae introduced onto mice (62.7%) than our previous study [17] (55.1%). Unlike the previous study, the moats were searched for non-engorged larvae on day 0 of post-tick attachment, which yielded a significant number of larvae. Within treatment and control groups, most non-engorged larvae were recovered within the first 24–48 h after tick introduction. Larval recovery was greater within the control groups (69.8%) relative to treatment groups (55.7%) which was likely a byproduct of the significantly greater likelihood of repletion and detachment within the control groups. It should also be noted that, while the adhesive mixture is useful for securing the capsules to mice, some larvae become trapped in it. While this did not affect the ability of most larvae within the capsules to feed on mice, it is worthy of discussion, and researchers should continue to refine methodology to further improve attachment success for larvae introduced onto mice.

The CP values were reduced within the reduced exposure group from an average of 191.5 ng/ml (days 1–5) to 29.4 ng/ml (days 15–19). This is expectedly lower than what was observed during our previous study [17] in which 48-h no-choice exposure resulted in average CP of 948.9 ng/ml (day 1) and 79.4 ng/ml (days 15–19). The only mouse which yielded larvae at day 15 in the current study did not have CP detectable > LOQ. The results of the other mice suggested that 100% control was obtainable when CP detectable at ≥ 4.4 ng/ml. This is encouraging, considering another study evaluating fluralaner as an oral acaricide concluded no significant treatment efficacy in groups where the fluralaner CP averaged 579 ng/ml (3% efficacy) and 208 ng/ml (4% efficacy) [38]. The results of reduced exposure suggested that extended exposure durations are not a necessity to deliver adequate fipronil bait to a feeding mouse. The benefit of extended exposure durations would be primarily to ensure maximum deliverability to wild mouse populations, including for individual mice to return regularly to a bait station and thus boost their fipronil levels. The average CP within the extended exposure groups was 10.6 ng/ml (day 21) and 1.0 ng/ml (day 35). A single mouse with CP > LOQ (4.8 ng/ml) yielded a single larva which did not molt, suggesting the LOQ is a reasonable indicator of the fipronil concentrations necessary to control 100% of feeding larvae. The proportion of treatment group mice having fipronil plasma concentrations < LOQ (1.25 ng/ml) increased at each subsequent time point: 0% (day 1), 10% (day 15), 40% (day 21), and 89% (day 35). Contrarily, while not 100% effective, results indicated that fipronil bait was still controlling larvae at day 35 post-exposure, indicating that fipronil could still be at least partially effective at concentrations < LOQ. We should note that the one mouse in T168-35 which had CP detectable > LOQ (8.8 ng/ml) was the only mouse within T168-35 which had 0 replete larvae collected from it. Future research should consider lowering the LOQ < 1.25 ng/ml to gain more explicit understanding of the concentrations required to eliminate larvae.

An oral acaricide bait targeting immature blacklegged ticks on white-footed mice could provide an economically advantageous control strategy. To best optimize the efficacy of this intervention strategy, it is important to consider the optimum time of year to apply the bait under field conditions. The majority of blacklegged tick larvae hatch out and feed during the summer [39]. Thus, targeted larval control could be conducted during the summer months. However, considering the crucial role of nymphs in the enzootic transmission cycle of *B. burgdorferi* s.s., it could be advantageous to also apply the bait in the spring when blacklegged tick nymphs are most abundant in order to reduce pathogen transmission to naïve hosts capable of serving as spirochete reservoirs [11, 39–42]. Prior researchers have deployed bait stations containing a topical tick control formulation (SELECT TCS^®^ bait boxes; Tick Box Technology Corp., Norwalk, CT, USA) in the summer and spring to control larvae and nymphs, respectively [43–46]. Preliminary laboratory data conducted under similar conditions to the current research indicate that our fipronil bait formulation is also effective against the nymphal stage of the blacklegged tick. Placing bait boxes both during spring and summer to control nymphs as well as larvae could be beneficial as the spring application would reduce transmission from *B. burgdorferi* s.s.-infected nymphs to susceptible rodents and the summer application would then ensure that larvae feeding on remaining infected rodents would not be able to feed to completion and molt to nymphs. This bait box application scheme is expected to reduce the density of host-seeking *B. burgdorferi* s.s.-infected nymphs in the spring of the following year. Field trials are needed to optimize the application scheme for delivery of an oral fipronil-laced bait to rodents in order to maximize the suppression of infected nymphal ticks posing a risk to humans.

## Conclusions

A low-dose fipronil bait, presented orally to white-footed mice at reduced and extended durations, controlled larval blacklegged ticks at days 1, 15, 21, and 35 post-exposure. It significantly reduced the number of larvae successfully feeding to repletion, detaching, and molting to nymphs. The study expands upon the results of a previous study [17] by more accurately simulating a variety of field scenarios in which mice might interact with one another and be exposed to alternative food sources. These results provide further insights into the potential of an oral fipronil bait to be used in Lyme disease prevention efforts.

## Supplementary Information

**Additional file 1: Table S1.** The specific timing of acclimation, exposure, post-exposure, tick attachment, and post-tick attachment for each test group.

## Data Availability

The data sets generated during and/or analyzed during the current study are available from the corresponding author upon reasonable request.
